# Prescription trends at the end of life in a palliative care unit: observational study

**DOI:** 10.1186/s12904-022-00954-z

**Published:** 2022-05-04

**Authors:** Tatiana Peralta, Maria Margarida Castel-Branco, Paulo Reis-Pina, Isabel Vitória Figueiredo, Marília Dourado

**Affiliations:** 1 Faculty of Medicine, University of Coimbra, Coimbra, Portugal; 2 Pharmacology and Pharmaceutical Care Laboratory, Faculty of Pharmacy, University of Coimbra, Coimbra, Portugal; 3 Institute for Clinical and Biomedical Research (iCBR), Faculty of Medicine, University of Coimbra, Coimbra, Portugal; 4 Palliative Care Unit “Bento Menni”, Casa de Saúde da Idanha, Sintra, Portugal; 5 Faculty of Medicine, University of Lisbon, Lisbon, Portugal; 6 Center for Studies and Development of Continuous and Palliative Care (CEDCCP), Faculty of Medicine, University of Coimbra, Coimbra, Portugal; 7 Centre for Health Studies and Research of the University of Coimbra (CEISUC), Faculty of Medicine, University of Coimbra, Coimbra, Portugal

**Keywords:** Drug prescription, Prescription trends, Deprescribing, Palliative care, End of life care, Hospice care

## Abstract

**Background:**

Symptomatic control is essential in palliative care, particularly in end-of-life, in which the pathophysiological changes that characterize this last phase of life strengthen the need to carry out an early therapeutic review. Hence, we aim to evaluate the prescribing pattern at a palliative care unit at two different time points: on admission and the day of the patient’s death.

**Methods:**

Quantitative, analytic, longitudinal, retrospective and observational study. Participants were adult patients who were admitted and died in a palliative care unit, in Portugal. Sociodemographic, clinical and pharmacological data were collected, including frequencies and routes of administration of schedule prescribed drugs and rescue drugs, from the day of admission until the day of death.

**Results:**

115 patients were included with an average age of 70.0 ± 12.9 years old, 53.9 were male, mostly referred by the Hospital Palliative Care Support Teams. The most common pathology was cancer, mainly in advanced stage. On admission, the median scheduled prescription was seven and “as needed” was three drugs. On the day of death, a decrease of prescriptions was observed. Opioids were always the most prescribed drugs. Near death, there was a higher tendency to prescribe butylscopolamine, midazolam, diazepam and levomepromazine. The most frequent route of drug administration was oral on admission and subcutaneous on the day of death.

**Conclusions:**

Polypharmacy is a reality in palliative care despite specialist palliative care teams. A reduction of prescribed drugs was verified, essentially due less comorbidity-oriented drugs. Further studies are required to analyse the importance of Hospital Palliative Care Support Teams.

## Background

Palliative Care (PC) is an integral, multidisciplinary, yet specialized medical care. PC focus is on relieving patients’ symptoms and improving the quality of life of both patients and their families when facing progressive, incurable, or life threatening diseases [1, 2]. Knowing that PC has a positive impact on the relief of suffering, comfort, and quality of life of all involved, PC should be offered as early as possible to all who can benefit from it [1]. In Portugal, as in other countries, PC needs are expected to continue to increase as the population ages [1]. The National Palliative Care Network, approved since 2012, was developed in a collaborative and integrated model involving the three healthcare levels of the National Health Service (Primary Healthcare, Hospital Healthcare and Integrated Continuous Care) [1].

PC is of great importance in the terminal phase of illness, i.e., during the last 3–6 months of life when symptom control and quality of life are essential [3–5]. Thus, it is necessary to identify symptoms early and treat them rigorously with multiple drugs. However, prescription in PC has some peculiarities arising from the physiological changes that the end-of-life (EOL) human body goes through. There are pharmacokinetic and pharmacodynamics modifications that change the risk/benefit ratio of prescription [3–6].

Moreover, the advanced stage of most pathologies, in an increasingly aged population with several comorbidities is a factor that contributes to the complexity of patients. In this context, polypharmacy (understood as the simultaneous taking of five or more drugs) is common and is often associated with the occurrence of pharmacological interactions and adverse drug events [7, 8].

When death is imminent it is necessary to simplify and withdraw unnecessary drugs, to optimize outcomes and reduce risks [7, 9–13]. In addition, it is desirable to increase the prescription of drugs used in controlling symptoms, relief of suffering, and providing comfort [7, 14, 15]. The major outcome becomes quality of life and avoid polypharmacy [3, 5–7, 15]. Extending life or preventing disabilities becomes secondary [3].

The objective of this study was to analyse the prescription pattern in a palliative care unit (PCU), at two different time points: 1) on admission (prior to the PCU team intervention) and 2) on the day of death. A secondary objective was to identify the most prescribed subgroup of drugs, the prescription prevalence, and the most used routes of administration.

## Material and methods

### Type of study and sample

This was a quantitative, analytic, longitudinal, retrospective and observational study. It included all adult patients admitted to the Poverello’s PCU in Braga, Portugal between August 1st, 2017 and December 31st, 2018. We excluded patients discharged to other institutions or home as well as patients with absent or incomplete clinical information.

### Definition of the variables and methods

Data extracted from clinical records included gender, age, provenance, diagnosis, number of comorbidities, functional status (using the Palliative Performance Scale - PPS) and the length of stay (in days) at the institution. We collected data regarding prescription at admission (T0) and on the day of death (T1), i.e., the number of prescribed drugs at fixed intervals of time or “scheduled prescription” (SP) and pro re nata “as needed” (PRN). The name of the drug (according to the international common name), the pharmacological subgroup (up to the second subgroup of the Anatomical Therapeutic Chemical Code - ATC), and the route of drug administration were also collected.

### Statistical analysis

Categorical variables were expressed with absolute and relative frequencies: n (%). Continuous variables were presented as mean and standard deviation (SD) or median and interquartile interval, Mdn (Q1,Q3). The normality of continuous variables was assessed by the Kolmogorov-Smirnov test. The McNemar test was used for comparisons between pharmacologic subgroups (SP and PRN) and prescribed drugs at T0 and T1. Chi-square tests for categorical variables were used to check for associations. The routes of administration were compared using a z score test for two population proportions. The Wilcoxon test was used to compare paired continuous variables. The software IBM-Statistical Package for the Social Sciences (v24) was used, and statistical significance was set at *p* < 0.05 (two sided).

## Results

During the study period, 145 patients were admitted to the PCU. We excluded 26 patients who were discharged and four who had unavailable prescription information. Hence, 115 patients were included. The average age was 70.0 ± 12.9 years old; 53.9% were male. Referrals to the PCU were done mostly by the Hospital Palliative Care Support Teams (90.4%). Cancer was the most frequent diagnosis (84.3%) mainly in advanced stage (56.7%) and predominantly lung cancer (20.6%). Most patients (73.9%) had two or more comorbidities and persistent complex needs. Most patients (88.8%) had major functional limitations with reduced mobility, need for assistance in basic daily activities, oral route limitations, and periods of altered state of consciousness (PPS < 50) (Table 1). Half of the patients were admitted in the last 2 weeks of life. The median length of stay in the PCU was 10 (5; 33) days, and 13.0% died in the first 48 hours after admission.

**Table 1:** Characteristics of the patients included in the study (*n* = 115)

Characteristics	*N* = 115
**Age** (years), mean ± SD	70.0 ± 12.9
**Length of hospitalization** (days), Mdn (Q1;Q3)	10 (5; 33)
**Gender,** n (%)	
Male	62 (53.9)
Female	53 (46.1)
**Provenance,** n (%)	
HPCST	104 (90.4)
CCICT	11 (9.6)
**Primary diagnosis,** n (%)	
Cancer	97 (84.3)
Heart failure	5 (4.3)
COPD	3 (2.6)
Ulcers	3 (2.6)
Renal failure	2 (1.7)
Other	5 (4.5)
**Location primary cancer,** n (%)	
Lung	20 (20.6)
Stomach	11 (11.3)
Colorectal	11 (11.3)
Central nervous system	9 (9.3)
Head and Neck	6 (6.1)
Esofagus	6 (6.1)
Pancreas	6 (6.1)
Liver	4 (4.1)
Uterus	4 (4.1)
Other	20 (20.6)
**Extension of disease,** n (%)	
Metastatic	55 (56.7)
Locally advanced /unknown	42 (43.3)
**Co-morbid conditions,** n (%)	
1	20 (17.4)
2 to 4	64 (55.7)
> 4	21 (18.2)
None/ unknown	10 (8.7)
**PPS,** n (%)	
≤ 20	29 (27.1)
30	29 (25.2)
40	37 (32.2)
50	11 (9.6)
60	1 (0.9)

*SD* Standard deviation, *Mdn* Median, *Q1* first quartile, *Q3* third quartile, n (%) number (percentage), *HPCST* Hospital Palliative Care Support Teams, *CCICT* Continuity of care/Integrated Care Teams, *COPD* Chronic obstructive pulmonary disease, *PPS* the Palliative Performance Scale

### Characterization of drug prescription

#### Quantitative analysis

Considering the total of prescriptions (T0 + T1), 167 different drugs were prescribed making up to 2270 prescriptions. There were 65 different pharmacological subgroups; T1 drugs from 20 identified subgroups were deprescribed, e.g., ATC A07A, B03B, G03A, M03B, and C09C. In SP, patients were prescribed a median of 7 (5; 10) drugs at T0 decreasing to 4 (3; 7) drugs at T1 (*p* < 0.001). In PRN, the median prescription increased from 3 (2; 4) to 4 (3; 5) drugs (*p* < 0.001) (Table 2).

**Table 2:** Description of schedule prescribed drugs and “as needed” drugs (*n* = 115)

Number of drugs	T0	T1	***p-value***
**Schedule prescribed drugs,** Mdn (Q1,Q3)	7 (5; 10)	4 (3; 7)	**< 0.001**^**a, b**^
**“As needed” drugs,** Mdn (Q1,Q3)	3 (2; 4)	4 (3; 5)	**< 0.001**^**a, b**^

T0- at admission. T1- the day of death. Mdn- Median. Q1 – first quartile. Q3- third quartile

^a^Wilcoxon test for paired samples

^b^statistically significant at 5%

#### Qualitative analysis

Opioids analgesics were the most prescribed subgroup in SP both at T0 (73.0%) and at T1 (82.6%) with a statistically significant increase (*p* = 0.013) (Table 3).

**Table 3:** Most common schedule prescribed drug subgroup at admission and the day of death

ATC code	Drug subgroup	Scheduled prescription		***p-value***^***a***^
		T0	T1	
		n (%)	n (%)	
N02A	Opioid analgesics	84 (73.0)	95 (82.6)	**0.013**^**b**^
A02B	Drugs for peptic ulcer and gastro-oesophageal reflux disease	77 (67.0)	25 (21.7)	**< 0.001**^**b**^
A06A	Drugs for constipation	64 (55.7)	29 (25.2)	**< 0.001**^**b**^
N06A	Antidepressants	48 (41.7)	21 (18.3)	**< 0.001**^**b**^
H02A	Corticosteroids for systemic use, plain	46 (40.0)	52 (45.2)	0.392
N03A	Antiepileptics	43 (37.4)	18 (15.7)	**< 0.001**^**b**^
N05A	Antipsychotics	41 (35.7)	34 (29.6)	0.265
B01A	Antithrombotic agents	41 (35.7)	11 (9.6)	**< 0.001**^**b**^
N05B	Anxiolytics	34 (29.6)	14 (12.2)	**< 0.001**^**b**^
A03F	Propulsives	38 (33.0)	22 (19.1)	**0.017** ^**b**^
C03C	High-ceiling diuretics	29 (25.2)	23 (20.0)	0.307
N05C	Hypnotics	21 (18.3)	35 (30.4)	**0.016** ^**b**^
C07A	Beta blocking agents	17 (14.8)	7 (6.1)	**0.006** ^**b**^
A03B	Belladonna and derivatives, plain	15 (13.0)	60 (52.2)	**< 0.001**^**b**^
C09A	Angiotensin-converting enzyme inhibitors, plain	7 (6.1)	1 (0.9)	**0.031** ^**b**^
A10A	Insulins and analogues	6 (5.2)	5 (4.3)	1.000
C03D	Potassium-sparing agents	6 (5.2)	4 (3.5)	0.727
C10A	Lipid modifying agentes, plain	6 (5.2)	1 (0.9)	0.063
A10B	Blood glucose lowering drugs, excl. Insulins	5 (4.3)	2 (1.7)	0.250
C09C	Angiotensin II receptor blockers, plain	4 (3.5)	0	–
C08C	Selective calcium channel blockers with mainly vascular effects	3 (2,6)	1 (0,9)	0,625
C08D	Selective calcium channel blockers with direct cardiac effects	2 (1.7)	0	–
C02A	Antiadrenergic agentes, centrally acting	1 (0.9)	0	–
C03B	Low-ceiling diuretics, excl. Thiazides	1 (0.9)	1 (0.9)	1.000

T0- at admission. T1- the day of death. ATC- the Anatomical Therapeutic Chemical Code

^a^McNemar Test

^**b**^statistically significant at 5%

Antispasmodics (A03B) were the second most prescribed subgroup in T1. From T0 (13.0%) to T1 (52.2%), we found a significant increase in its prescription (*p* < 0.001). Corticosteroids, were the third most prescribed class at T1. They increased from 40.0% (T0) to 45.2% (T1) without statistical significance (*p* = 0.392). We found that 84.8% of patients undergoing corticosteroid therapy at T0 were treated concomitantly with antiulcer agents (*p* = 0.001). At T1, no statistical association was found between anti-ulcers agents and corticosteroid prescription (*p* = 0.054).

Laxatives (A06A) were the third most prescribed subgroup in T0 that decreased from 55.7% (T0) to 25.2% (T1) with statistically significant (*p* < 0.001). No association was found between opioid and laxatives prescription in SP [*p* = 0.190 (T0) and *p* = 0.678 (T1)] nor between opioid in SP and laxatives in PRN prescription [*p* = 0.194 (T0) and *p* = 0.134 (T1)].

Regarding anti-dyslipidemiants, including statins, we found 5.2% of prescription in T0, having been deprescribed in 83.3% of patients in T1.

Hypoglycaemic agents (A10A and A10B) were withdrawn in both regimes, and statistical significance was found only in PRN [14.8% (T0), 6.1% (T1); *p* = 0.031].

As to drugs with antihypertensive potential (subgroups ATC C02A, C03B, C03C, C03D, C07A, C08C, C08D, C09A and C09C), there was a statistically significant reduction in the prescription [38.3% (T0) to 27.0% (T1), *p* < 0.029]. The proportion of antithrombotic agents prescribed reduced from 35.7 (T0) to 9.6% (T1) (*p* < 0.001). The use of antidepressants decreased significantly from 41.7% (T0) to 18.3% (T1) (*p* < 0.001).

Considering PRN prescription, opioids were the most prescribed subgroup with statistical difference between T0 (76.5%) and T1 (92.2%; *p* = 0.001) (Table 4). Analgesics and antipyretics represent the second most prescribed subgroup in T0 (45.2%). At T1, antipsychotics were the second most prescribed class with a significant increase in prescription compared to T0 [20.0% (T0), 64.3% (T1), *p* < 0.001]. Propulsive, i.e., metoclopramide, were prescribed upon 25.2% (T0) and 38.3% (T1), with a statistically significant difference (*p* = 0.032).

**Table 4:** Most common “As Needed” drug subgroup at admission (T0) and the day of death (T1)

ATC-code	Drug subgroup	Prescription “as needed”		***p-value*** ^***a***^
		T0	T1	
		n (%)	n (%)	
N02A	Opioid analgesics	88 (76.5)	106 (92.2)	**0.001**^**b**^
N02B	Non-opioid analgesics and antipyretics	52 (45.2)	57 (49.6)	0.552
A06A	Drugs for constipation	41 (35.7)	34 (29.6)	0.324
A03F	Propulsives	29 (25.2)	44 (38.3)	**0.032**^**b**^
N05C	Hypnotics	24 (20.9)	32 (27.8)	0.215
N05A	Antipsychotics	23 (20.0)	74 (64.3)	**< 0.001**^**b**^
A10A	Insulins and analogues	17 (14.8)	7 (6.1)	**0.031**^**b**^
N05B	Anxiolytics	16 (13.9)	43 (37.4)	< 0.001
A04A	Antiemetics and antinauseants	9 (7.8)	5 (4.3)	0.289
A03B	Belladonna and derivatives, plain	4 (3.5)	25 (21.7)	**< 0.001**^**b**^
A02B	Drugs for peptic ulcer and gastro-oesophageal reflux disease	2 (1.7)	0	–
C09A	Angiotensin-converting enzyme inhibitors, plain	2 (1.7)	1 (0.9)	1.000
M01A	Antiinflammatory and antirheumatic products, non-steroids	1 (0.9)	2 (1.7)	1.000
B02A	Antifibrinolytics	1 (0.9)	5 (4.3)	0.219
A07D	Antipropulsives	0	2 (1.7)	–

T0- at admission. T1- the day of death. ATC- the Anatomical Therapeutic Chemical Code

^a^McNemar Test

^**b**^statistically significant at 5%

**Fig. 1:**
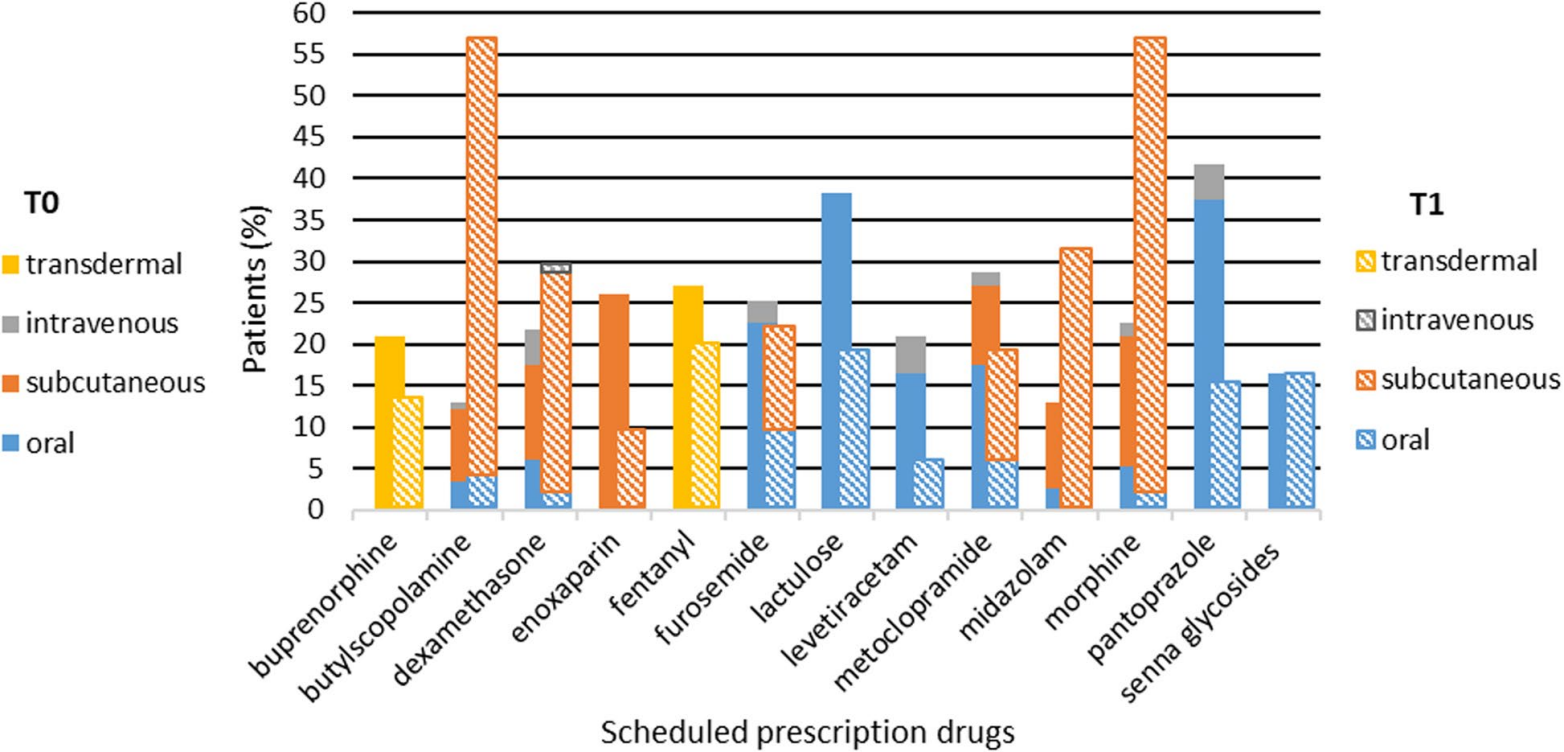
Top individual scheduled prescription at admission (T0) and the day of death (T1) and routes of administration

**Fig. 2:**
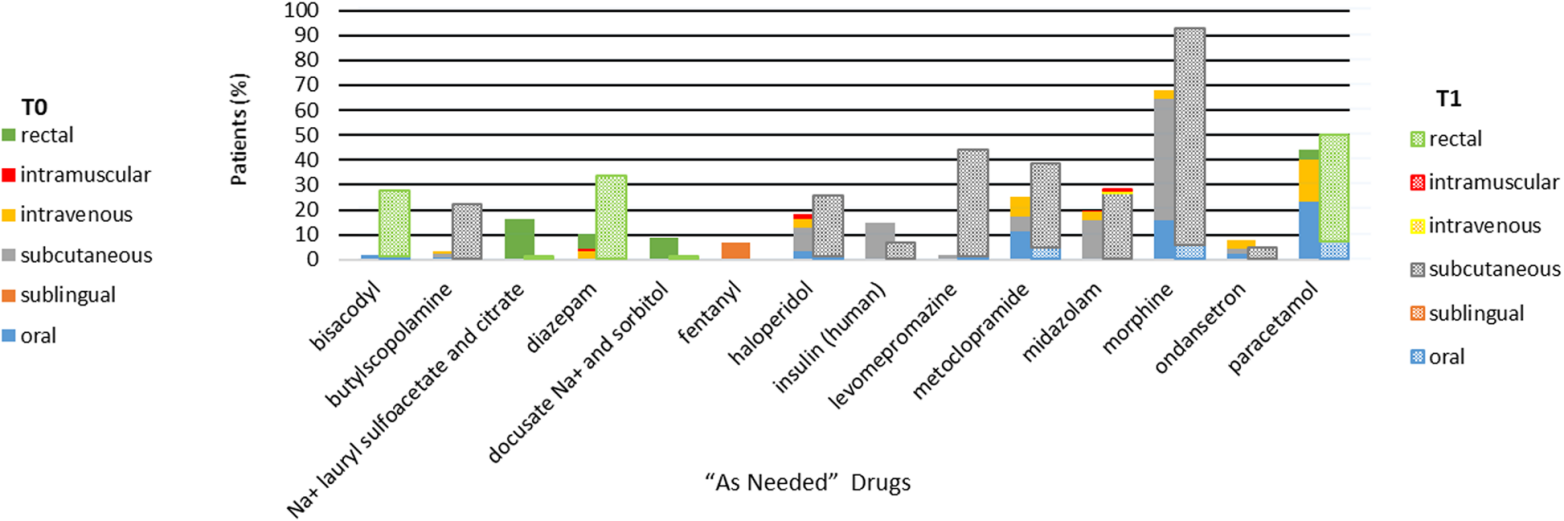
Top individual “As Needed” prescription at admission (T0) and the day of death (T1) and routes of administration

The individual drugs prescriptions are summarized in Figs. 1 and 2.

Regarding individual SP, most drugs were deprescribed from T0 to T1 with a statistically significant decreased for levetiracetam (*p* < 0.001), enoxaparin (*p* < 0.001), metoclopramide (*p* = 0.017), lactulose (*p* < 0.001), and pantoprazole (*p* < 0.001). We found a statistical prescription increased in morphine (*p* < 0.001), butylescopolamine (*p* < 0.001), and midazolam (*p* = 0.001).

For PRN, we found a statistically significant increase in the prescription of morphine (*p* < 0.001), metoclopramide (*p* = 0.032), diazepam (*p* < 0.001), butylescopolamine (*p* < 0.001), levomepromazine (*p* < 0.001), and bisacodile (*p* < 0.001). There was a statistically significant decrease between T0 and T1 in the prescription of docusate Na^+^ and sorbitol (*p* < 0.001), insulin (human) (*p* = 0.031) and Na^+^ laurylsulfoacetate and citrate (*p* < 0.001).

#### Administration routes

At T0, medication was administered mostly orally either in SP (70.3%) or in PRN (28.7%). At T1, there was a significant decrease in the use of oral route, which in SP decreased to 41.6% (*p* < 0.001) and in PRN to 7.2% (*p* < 0.001).

In T0, the intravenous route was used in both SP (4.8%) and PRN (16.7%). We found a decrease in the use of this route in T1 both in SP (0.3%) and in PRN (0.7%).

The subcutaneous route increased at T1 both in SP [from 12.4% (T0) to 47.3% (T1), *p* < 0.001] and in PRN [from 36.9% (T0) to 64.7% (T1), *p* < 0.001].

The continuous perfusion by the subcutaneous route was used at T0 in 7.0% and at T1 in 40.9% of patients. At SP, there was subcutaneous administration of metoclopramide, dexamethasone, morphine, butylscopolamine, and furosemide. Concerning PRN, the subcutaneous route was used to administer haloperidol, butylscopolamine, ondansetron, metoclopramide, and morphine. Figs. 1 and 2 show remaining routes of administration and individual drugs.

## Discussion

### Quantitative analysis

This study demonstrated that polypharmacy was present at EOL although there was a tendency to reduce the prescription of regular fixed drugs and increase of rescue drugs.

With no consensus on the issue, polypharmacy is usually defined as the simultaneous use of multiple drugs (frequently more than five) [8]. Side effects can occur in up to 80% of people when seven or more drugs are prescribed [14, 15]. We found that polypharmacy was present in more than half of the patients studied. On admission, patients had a median of seven schedule prescribed drugs and three “as needed” drugs. The median of schedule prescribed drugs at admission and at day of death was consistent with the existing literature [14, 16–19].

A reduction of drugs prescriptions from admission to death was verified, essentially due to the decrease in drugs aimed to treat or control comorbidities-some of these are considered potentially inappropriate [17, 18, 20–22]. Potentially inappropriate drugs were defined as drugs with increased risk for adverse events that outweigh eventual benefits, prevention of illnesses without short-term benefit, or conflicts with individual patient’s care goals [9, 20, 21]. Most patients were referred from Hospital Palliative Care Support Teams, a specialist palliative care team, that provide support and expert advice to complex situations [1]. Despite specialist palliative care consultation, polypharmacy continues to be an issue that might be explained to limitations related to human resources, training level, level of integration and development of palliative care programmes and survival prognostication [1, 23–25].

Deprescribing is a complex process that requires a careful assessment of the benefits and harms for patients and requires shared decision-making between doctors, patients, and pharmacists, which is not always easy [4, 12, 18, 21, 22, 26]. Frequent and systematic re-evaluation of the adequacy and safety of the prescription over time- as well as the appropriateness and effective cessation- are required [3, 13, 20, 21].

The significant increase observed in “as needed” prescription from admission to death was explained by the increased prescription of analgesics and antipsychotics [22]. This is consistent with other studies such as Sera et al. who reported 7.9 per patient “as needed” drugs used for pain relief, delirium, and anxiety for patients in a PCU [15, 27, 28].

### Qualitative analysis

Pharmacological subgroups considered potentially inappropriate include dyslipidaemic drugs (particularly statins). Here, we found a lower prescription proportion than other studies at admission (5% vs 29%); most of them were withdrawn [16, 18, 27, 29]. Statins are not considered useful in patients with limited life expectancy and at EOL [18]. These drugs can be associated with several problems such as myopathy, myalgia, liver dysfunction, and acute renal failure among others. Some multicentre studies have shown that there are no benefits to cardiovascular prevention when life expectancy is limited [30, 31]. Therefore statins withdrawal is safe and is associated with a significant improvement in quality of life [30–33].

We found a significant deprescription of antihypertensive drugs that could suggest that these drugs might be futile at this stage [6, 18]. However, this subgroup includes diuretics drugs such as furosemide that are widely used in PC for symptomatic control [2].

Antithrombotic agents were also significantly deprescribed with a preference to low-molecular weight heparin, mainly enoxaparin, both at admission and at the day of death. Research regarding the risk of thrombosis in EOL is scarce however the administration of heparin seems to have no impact on patient survival [32, 34–36].

We found that antiulcer drugs were prescribed both at admission (68.9%) and at death (21.8%) more than what was found in other studies [14, 18, 27, 34]. In our study, patients with corticosteroid are more likely to have anti-ulcer agents at admission. Prescription appropriateness such as history of gastrointestinal bleeding, peptic ulcer, gastritis, or chronic use of non-steroidal anti-inflammatory drugs for more than 30 days has not been assessed. In PC setting, antiulcer drugs can be considered futile upon 50% of prescriptions [16, 18, 27, 32].

Diabetic treatment in patients in EOL can conflict with quality of life due to injections and glycaemic control that can explain the withdrawal of “as needed” hypoglycaemic drugs. We have not assessed hyperglycaemia symptoms [37–39].

Metoclopramide is a propulsive drug and is recommended for the first line management of nausea and vomiting explaining the increased “as needed” prescription observed at day of death [2, 40].

Butylscopolamine, was one of the most prescribed drugs at day of death in both prescription regimes. This anticholinergic drug relaxes smooth muscles and reduces secretions from the respiratory and digestive tract, which helps to control some of the most prevalent symptoms in PC such as nausea and vomiting, pain, respiratory tract secretions, and intestinal occlusion [2, 41].

Common symptoms at EOL include anxiety, depression and sleep disturbance [2]. The prescription of antidepressant decreased significantly. In this study, in the subgroup of anxiolytics, diazepam was the most “as needed” prescribed mainly by rectal administration. This is explained by the incompatibility with subcutaneous route of administration. In the hypnotic/sedative subgroup, midazolam was the most prescribed in both moments and prescription regimes. Unlike other anxiolytics, midazolam can be administered subcutaneously or intravenously with a rapid onset of action. It is used as a sedative and in seizures. Neuroleptics/antipsychotics are useful to control several symptoms such as anxiety, psychomotor agitation, and delirium/confusion [2]. Haloperidol is a first choice for agitation associated with delirium in EOL. It was highly prescribed “as needed” at admission and day of death [40, 41]. It can also be used for anxiety refractory to benzodiazepines, presence of psychotic symptoms, or secondary anxiety caused by corticosteroids. Haloperidol, levomepromazine and midazolam are essential drugs used in sedation including palliative sedation when refractory symptoms are present near death such as massive terminal haemorrhage, asphyxiation due to respiratory obstruction, or uncontrollable pain [2, 41].

Pain is one of the most impacting symptoms for patients, their families, and caregivers. It has multidimensional and multisystem manifestations inducing a loss of quality of life [2]. In this study, opioid and non-opioid analgesics were the most prescribed drugs on both regimes and moments. At day of death, a statistically significant increase in opioid prescription was found, which is related to the need to ensure adequate pain control. Pain management requires a multimodal approach and might need adjuvant drugs such as corticosteroids, anticonvulsants, and benzodiazepines [2]. In this study, the use of those drugs increased at the day of death.

Constipation has a multifactorial aetiology and can affect quality of life [2]. It is recommended that laxatives must be prescribed to patients receiving opioids, however we found no association between opioid and laxative prescription [2, 40].

### Administration routes

Most patients receiving intravenous drugs at admission came from the hospital.

In EOL, the presence of vomiting, nausea, gastric stasis, dysphagia, as well as changes in the state of consciousness, intestinal obstruction, or others limits the use of oral administration and justifies the high frequency of subcutaneous prescription [2, 18]. The subcutaneous route is a suitable and effective route with fewer risks of local and systemic complications. Not all drugs can be administered subcutaneously according to the summary of their drug characteristics such as furosemide, levomepromazine, metoclopramide, dexamethasone, midazolam, and haloperidol. Off-label prescription is one of the main challenges of prescribing in PC that must be standardized in order to make the work of the PC teams safer [1].

Several limitations are known. First, this study is a retrospective and single-unit study. Second, the clinical appropriateness of prescribing practices such as indication or effectiveness has not been assessed. The time between referral to admission and the disease progression time were also not considered. Finally, the type of symptoms, suffering, and quality of life were not evaluated.

## Conclusion

Potentially inappropriate drugs are common in patients referred to PC units. Deprescription can be improved in Hospital PC Support Teams. From admission until death, there was a reduction in the scheduled prescription drugs for related comorbidities and an increase in the number of rescue drugs used for symptomatic control. The subcutaneous route was the preferred route of administration at the time of death. Opioids were the most frequent subgroup prescribed. Off-label prescription is one of the challenges in PC prescribing. Despite these limitations, our study describes current prescription patterns in PC and demonstrates the complexity of EOL prescription. Efforts should be made into raising awareness of deprescription especially in the hospital setting and the focus on symptomatic control at the EOL.

## Data Availability

The datasets used and analysed during the current study are available from the corresponding author on reasonable request.

## Abbreviations

ATC: Anatomical Therapeutic Chemical code
EOL: End of life
PC: Palliative care
PCU: Palliative care unit
PPS: Palliative Performance Scale
PRN: Pro re nata (as needed) prescription
SP: Scheduled prescription
